# Waste Reduction and Management in Food Security and Food Sustainability

**DOI:** 10.3390/foods15040749

**Published:** 2026-02-18

**Authors:** Ali Khoddami

**Affiliations:** Sydney Institute of Agriculture, School of Life and Environmental Sciences, Faculty of Science, The University of Sydney, Sydney, NSW 2006, Australia; ali.khoddami@sydney.edu.au

## Introduction

Climate change—one of the major global challenges—significantly affects agricultural productivity, the accessibility of water resources, and the stability food systems. Increases in temperature, extreme weather phenomena, and variable rainfall patterns led to a rise in pest and disease pressures and a reduction in crop yields [1,2], limiting the accessibility of rich food resources. Due to the increase in the global population, evolving food preferences, and lifestyle changes, food production is expected to be higher in the next 35 years compared to that of the entire history of mankind [3]. It is, therefore, necessary to provide food security for the growing world population while promoting the use of natural resources in a sustainable manner. In this regard, food security—as a multidimensional concept—refers to the economic, social, and physical access of all people to nutritious and sufficient food, that meets their dietary needs and preferences, at all times. Consequently, promoting a culture of avoiding food waste and managing food sustainably in different countries is essential to address environmental challenges and to ensure food security.

According to the Food and Agriculture Organization of the United Nations [4], about one-third of global food production meant for human consumption leads to food waste, and it occurs at different levels of the food supply chain. These levels include agricultural production, post-harvest transportation, processing, distribution, retail, and consumption. The application of advanced technologies, along with interventions in consumer behavior, can be considered efficient solutions for countering food waste [5]. Food waste harms the environment in a number of ways, including deforestation, water waste, greenhouse gas emissions from organic matter decomposition in landfills, and energy consumption for manufacturing and transportation [6,7]. On the other hand, the conversion of organic waste into high-value bioactive compounds is possible; this approach is considered a sustainable solution to reduce waste and increase the efficiency of resources. Vitamins are often found among food waste, despite the fact that they can be reused in different food- and medicine-related products [8]. These materials may also be used as secondary resources to create food items with added value. The significantly high levels of food waste can be considered an opportunity to address food insecurity, combat climate change and environmental damage, as well as to save energy [9].

Furthermore, the management of food waste at the consumer level—including the management of relevant behaviors—is considered imperative to decrease food waste and reduce its environmental impacts. Despite expressing an aversion to food waste, consumers often engage in behaviors that lead to food waste [10]. This is especially prevalent among the younger generation—a key consumer group that shapes the future population and plays an important role in reducing this environmental problem. The following is an attempt to briefly present research that has addressed various challenges, such as food security, food waste, and consumer behavior, as well as sustainable solutions to reduce environmental impacts, in order to reduce food waste and wastage from farms to households, and prevent environmental degradation and economic inefficiency.

In the article “*Global Comparison and Future Trends of Major Food Proteins: Can Shellfish Contribute to Sustainable Food Security?*” Contribution 1 (2025) examine the environmental quality and food security related to food production. The work also analyzes innovative and environmentally friendly protein sources, such as lab-grown foods, microorganisms, and insects, as well as vegetables. This study employs a combined approach to integrate quantitative and qualitative data from the literature and public databases. The effects of various protein resources on agricultural land use and greenhouse gas emissions were evaluated, and the possibility of carbon sequestration within the supply chain was investigated. In addition, future trends regarding global protein resources were reviewed, and shellfish were introduced as a significant, environmentally friendly and nutritional option. It was reported that this achievement is possible via shell biocalcification, which maintains low-impact production and contributes to the reduction in climate change. Moreover, in order to evaluate consumer, environmental, and nutritional challenges, all main protein resources like vegetal proteins, farmed seafood, and terrestrial livestock—along with alternative sources like cultured cells and insects—were considered. Bivalve aquaculture is a zero-emissions method that is dependent on natural phytoplankton and free of additives or antibiotics, which can have a minimal environmental footprint. The results of the study suggest that Bivalve aquaculture, including scallops, clams, mussels, and oysters, has great potential to increase food security and promote sustainable protein consumption by decreasing land use and assisting in mitigating climate change, environmental impacts, and atmospheric carbon. Since the need for global food is expected to increase in the future, seafood aquaculture is predicted to expand due to its benefits to human health and its lower environmental impact. The authors believe that challenges need to be addressed to fully exploit the potential of bivalve aquaculture as a sustainable economic activity.

The article “*What Drives Generation Z to Avoid Food Waste in China? An Empirical Investigation*” by Contribution 2 (2025) examines food waste avoidance behaviors among Generation Z in China from a psychosocial perspective. Food waste avoidance not only contributes to a reduction in environmental pressure and resource waste but also assists in achieving the objectives of sustainable development. Because of the features of the consumption environment and the consumers of this generation, the Theory of Planned Behavior (TPB) is the foundation for this research. After the collection of 417 valid responses via online survey and a questionnaire, structural equation modeling was employed to analyze the data. The findings indicate that Generation Z’s intentions to prevent food waste are positively impacted by attitude, subjective norms, and perceived behavioral control. Additionally, attitudes and perceived behavioral control are positively and significantly impacted by moral self-identity. Furthermore, it is shown that scarcity mentality has a beneficial and moderating effect. The authors categorize the results of this paper into three areas (both theoretical and practical), including (I) the introduction of scarcity mindsets and moral identity leads to the extension of the TPB model and the deepening of our understanding about psychological mechanisms related to the avoidance of food waste; (II) experimental results indicate the TPB model’s robustness, which demonstrates complex correlations among variables, indicating the significant moderating impacts of scarcity mindsets and moral identity, and demonstrating how social influences affect behavioral intentions in particular contexts; and (III) PBC is often influenced by environmental constraints, and environmental amenities can allow Generation Z to manage food more effectively. According to the authors, researchers should be expected to investigate the actual, real-world food waste behaviors of this generation, rather than simply their intentions. Moreover, focusing on Generation Z leads to the lack of generalizability of the results. Furthermore, cross-national comparisons between Generation Z from various cultural origins may highlight notable differences in attitudes and behaviors and provide insightful information for the creation of successful international initiatives and policies.

The “*HRMS Characterization and Antioxidant Evaluation of Costa Rican Spent Coffee Grounds as a Source of Bioactive Polyphenolic Extracts*” by Contribution 3 (2025) presents a thorough analysis of Costa Rica’s spent coffee grounds (SCGs), which serve as an abundant source of secondary metabolites possessing antioxidant qualities. The study used high-resolution mass spectrometry (HRMS) and ultra-performance liquid chromatography (UPLC-QTOF-ESI MS) to test aqueous extracts collected from various SCG samples. A total of twenty-one substances were identified based on the data, including four atractyligenin diterpenes, three caffeoylquinic lactones, and fourteen phenolic acids. Additionally, the study assessed antioxidant activity using four distinct assay techniques: the Trolox equivalent antioxidant capacity (TEAC) assay, the oxygen radical absorbance capacity (ORAC) assay, the potassium ferricyanide-reducing antioxidant power (PFRAP) assay, and the 2,2-diphenyl-1-picrylhydrazyl (DPPH) assay. The results revealed that the fine-grind sample outperformed the medium- and coarse-grind samples in antioxidant evaluations and had the greatest amounts of total polyphenols and phenolic acids. The findings highlight the importance of SCGs in metabolites that confer health benefits; therefore, there is interest in developing functional and therapeutic food innovations, given their potential health advantages.

The article “*Up-Cycling Broccoli Stalks into Fresh-Cut Sticks: Post-harvest Strategies for Quality and Shelf-Life Enhancement*” Contribution 4 (2025) examines the use of broccoli stems as a discarded agricultural by-product, which have less value in the context of fresh consumption and are only offered to the market as broccoli florets. In this research, broccoli stems—as a fresh cut product with added value—were evaluated by employing post-harvest storage strategies for 11 days at 5 °C to maintain their quality and increase the shelf life of the product. A mixed methodology was followed in this study to examine various storage and treatment approaches. In addition, Statgraphics Centurion XV software was used to perform ANOVA. Regarding various post-harvest treatments, hot water treatment (HWT) was reported to reduce browning and increase shelf life by 11 days. This result indicated the control of respiration and growth of microbes since responses related to natural defense in plant tissues are stimulated by heat, which leads to a rise in the resistance to microbial invasion. This feature led to the achievement of the highest sensory quality scores while being less efficient in the preservation of bioactive compounds. A combination of treatment with HWT + calcium ascorbate (CaAsc) was reported to be most effective in the optimization of quality and preservation of compounds that play a role in health promotion. The antioxidant effect of ascorbic acid in CaAsc was a potential reason for this synergistic effect. A combination of calcium and ascorbic acid that significantly preserves vitamin C. Trehalose (TREH) alone did not show a positive preservative effect and caused browning, increased respiration, and microbial proliferation. Moreover, basic hygiene and storage practices ensure microbiological safety during storage, such as a lack of foodborne pathogens—including E. coli and Salmonella spp. After the comparison of different treatment approaches, the combined method based on antioxidant and mild thermal treatments was reported to be promising for the preservation of broccoli stems. This indicates the applicability of these stems as a valorization of food waste in addition to satisfying the principles of a circular economy.

The paper “*Optimizing Farmers’ and Intermediaries’ Practices as Determinants of Food Waste Reduction Across the Supply Chain*” by Contribution 5 (2025) focused on the optimization of pre- and post-harvest practices, as well as the interaction between economic and social traits of intermediaries and farmers for an increase in agri-food products’ shelf life. Some approaches were reported to lead to a reduction in food losses and the highest levels of final returns for quality. Because grape producers in Egypt are vulnerable to changes in markets (at global levels)— due to the lack of land fragmentation and marketing information among small producers—this study examined data from 343 grape farmers and intermediaries in Egypt. The work used a combination of Ordinary Least Squares (OLS) and Fractional Regression Models (FRM) to analyze strong correlations between social and economic characteristics and stakeholder practices at different food supply chain stages. Key findings show that practices such as drip irrigation (13.9%), avoiding the use of ripening chemicals (24%), increasing farmers’ cultivation area (6.1%), improved packaging (13.7%), and harvesting in the early morning hours (8.7%) can significantly reduce food waste. Moreover, for intermediaries, increasing the distance to market increased waste by 0.15%; whereas, employing suitable warehouses, participating in formal markets, and choosing the right transportation method reduced waste by 65.9%, 13.8% and 7.9%, respectively. Furthermore, food waste may be greatly decreased by combining these strategies. The authors additionally contend that post-harvest losses (PHL) are decreased by improving farmer knowledge and farmed area, and that formal agricultural markets help lower post-harvest losses by approximately 14% relative to informal markets. Furthermore, the decrease in PHL and the health of people and the environment were greatly enhanced by the lack of toxins. In order to lower PHL, the authors stress the need to integrate market types with suitable transportation and storage capabilities.

In the paper “*Moving Beyond Indices: A Systematic Approach Integrating Food System Performance and Characteristics for Comprehensive Food Security Assessment,*” the limits of food security indicators—such as possible ranking biases and insufficient understanding of food system dynamics—were studied by Contribution 6 (2025). Indicators of food security are used to make decisions and evaluate the ability of a nation to endure international economic and ecological crises. By using a methodology that incorporated two-step clustering and elastic-net regression-based feature selection, this study overcame these constraints and supported food system management by assisting decision-makers in implementing organized techniques for well-informed decision-making. This study used a new mathematical and operational approach using the Economist Intelligence Unit (EIU) dataset of 94 countries, selecting 15 indicators for the first model and 9 indicators for the second model from an initial set of 25 indicators. A two-stage clustering approach was then used to separate the countries into four categories according to a combination of income levels and food system features. This combined approach addressed the limitations of traditional input-based models, which place an emphasis on the availability of resources but do not link the composite indicator to food system outcomes. The nutritional condition of the population was taken into account as an essential output indicator in the input–output model, indicating the availability of daily food needs. According to the findings, countries like Switzerland and Germany that have high per capita incomes as well as integrated and industrialized food systems, which provide consistent food security for their people. However, food insecurity is observed more in traditional and rural food systems as well as in low-income countries. These systems could focus on policies that foster agro-technological innovation, increase access to agricultural finance, and enhance transport infrastructure. The suggested method cannot monitor changes or trends over time since it is only based on cross-sectional data.

The article *“Culinary Home Empowerment for Food Waste Prevention and Minimization: Feasibility and Efficacy Protocol”* by Contribution 7 (2024) outlines a protocol that assesses the feasibility, acceptability, and preliminary effectiveness of a household food-waste prevention and reduction program. The intervention features eight modules, each with three to five short videos, as well as downloadable recipes and worksheets. The impact of this program would be examined in two distinct contexts: (1) a population-based setting in which home cooks voluntarily access the content and (2) a community-based setting in which the content is delivered intensively as part of a remote class. At the population level, the study processed outcomes such as feasibility, usage, acceptability, and satisfaction, as well as preliminary efficacy outcomes, including motivation, opportunity, and ability. It employs a single-arm quasi-experimental post-test-only design, in which the viewers are asked to complete a brief five-item post-video survey. Additionally, a separate recruited sample would undergo a more detailed assessment, including pre- and multiple post-test surveys. At the community level, the mixed-methods evaluation assesses feasibility, usage, acceptability, and satisfaction, as well as preliminary efficacy outcomes including motivation, opportunity, and ability. It also considers additional outcomes such as household food waste and sustainable dietary practices. The authors believe that the proposed protocol can translate findings into prevention lessons to minimize food waste at the consumer level. In keeping with a protocol article, this article is presented without results with statistical analysis prior to data collection. In order to guide policy and investment for program interventions, the authors hope that data analysis will reveal elements of the intervention that can prevent and reduce waste more broadly, informing policy and investment decisions.

In the article “*Solid-State Fermentation of Cereal Waste Improves the Bioavailability and Yield of Bacterial Cellulose Production by a Novacetimonas sp. Isolate,*” the importance of cereal wastes like rice bran and cereal dust as by-products was examined by Contribution 8 (2024) using three mold isolates and a low-cost solid-state fermentation (SSF) pre-treatment. They also attempted to convert waste into a renewable, eco-friendly biomaterial that is crucial for the cost-effective and sustainable production of bacterial cellulose. The environmental effect of agro-industrial waste and the high cost of traditional culture media were demonstrated by substituting untreated rice bran or cereal dust for the culture medium. In contrast to the untreated control sample, the application of rice bran fermented with Rhizopus oligosporus produced a greater BC. In this work, when compared to Hestrin-Schramm (HS), the costs to produce culture medium decreased by up to 90%. The produced BC were characterized using derivative thermogravimetry (DTG), thermogravimetric analysis (TGA), X-ray diffraction (XRD), Fourier-transform infrared spectroscopy (FTIR), energy-dispersive X-ray spectroscopy (EDS), and scanning electron microscopy (SEM) techniques to gain insight into its chemical bonding, morphology, and internal structure. It was also found that there were comparable properties of biopolymer between the cellulose produced in waste-based and standard culture media, demonstrating that the SSF-based process can be effective in converting cereal waste to BC. The SEM technique demonstrated that the BC’s nanostructure, fiber diameter, and shape were impacted by both the drying process and the selection of fungal strain for SSF. However, the obtained results from FTIR and XRD tests verified that the cellulose I structure remained intact in bacterial cellulose derived from treated substrates via solid-state fermentation. The research also found that SSF treatment enhanced the thermal stability of BC made from rice bran and cereal dust.

The article “*Tackling Food Waste: An Exploratory Case Study on Consumer Behavior in Romania*” by Contribution 9 (2024) conducted an analysis of the factors influencing household consumer behavior regarding food waste (FW) in Romania. Their aim was to understand the motivations to reduce FW, and investigate attitudes towards FW and food consumption behaviors in households. Data was collected using an online, self-administered questionnaire from a non-probability sample in Romania to examine consumer behaviors related to food waste avoidance. Moreover, a stepwise linear regression model and descriptive statistical analyses were performed to evaluate the Romanian consumers’ behaviors to decrease FW as well as to specify the effect of various determinants on the motivation of consumers for avoiding FW. The findings point to important factors like the desire to lessen environmental effects; discussions about FW reduction in the home; established consumption patterns, particularly when cooking at home; the person’s social circle, and personal, social, and ecological responsibilities; and involvement in awareness campaigns. In fact, the authors emphasize that FW avoidance behavior emerges from family environment, that discussions in the household are valuable, and that by socializing new generations with this spirit there will be a significant impact on FW reduction. The research points out the importance of educational and awareness-raising strategies, which are essential for changing consumer attitudes and behaviors in Romania. Furthermore, the discussion includes a comparative analysis with the results of studies documented by researchers in other countries, which highlights the differences in FW avoidance behavior of individuals in different cultural, social, and economic environments. Due to the limited number of participants, this study does not have appropriate generalizability to the entire Romanian population.

In the article “*Antioxidant Bio-Compounds from Chestnut Waste: A Value-Adding and Food Sustainability Strategy*” Contribution 10 (2025) concentrated on the antioxidant qualities of certain chestnut burr cultivars, to address the possibilities of a circular bioeconomy via the value-adding of agricultural leftovers and the dual issues of waste management and public health. According to the findings, almost one-third of food production is wasted, which increases environmental and human concerns. Utilizing the circular bioeconomy, biological waste may be converted into useful goods that can be used in sustainable food production. Based on their phenolic content, this study evaluates the hydroalcoholic extract content and antioxidant qualities of three chestnut burr cultivars from Mount Amiata that are certified under Indicazione Geografica Protetta (PGI)—Marroni (RM), Cecio (RC), and Bastarda Rossa (RB). In vitro assays were performed to assess their significance in combating oxidative stress. Because of their antioxidant properties, the investigations indicated that gallic and ellagic acids are the most prevalent components in the extracts. The results also demonstrated that the peel extracts of the Cecio and Bastarda Rossa cultivars exhibited antioxidant activity and much greater amounts of important bio-compounds, such as flavonoids, gallic acid, and ellagic acid. Moreover, a cytotoxicity test confirmed that all three extracts are safe for human cell lines. The in silico docking simulations that were performed in the study demonstrated that the chemicals may spontaneously bind to predicted targets, such as proteins associated with oxidative stress. This work advances functional foods and emphasizes the importance of chestnuts in promoting practices related to the circular bioeconomy, which focuses on the production of value-added products for biological applications. This approach would help to reduce the environmental pollution caused by the incineration of chestnut waste.

In the article “*Quality Properties of Bakery Products and Pasta Containing Spent Coffee Grounds (SCGs): A Review*” Contribution 11 (2024) examined spent coffee grounds (SCGs) as a type of waste that can be used as a material to create new foods. They found that incorporating different percentages of SCGs provided different quality properties in bakery and pasta products. This study used a literature review, with a comprehensive search of reputable databases and content analysis of published studies, to investigate the effects of adding different percentages of SCGs to different products. The study was conducted on baked products such as pasta, muffins, cakes, biscuits, bread, and cookies. The authors highlighted that, from a chemical point of view, SCGs contributed to increasing the nutritional value (protein, fat, fiber, ash, and antioxidant content) and health benefits of the baked products. They also emphasized that SCGs affected the physical properties of the products and that the effect depended on the concentration of SCGs, the coffee variety, and the processing method. The authors also investigated the effect of SCGs on the sensory properties of the products. The incorporation of SCGs improved their sensory properties, adding a specific flavor and color to the baked goods. It is extremely important that the microbiological safety of pasta and bread, which guarantees the safety and quality of the product for human consumption, is not compromised when SCGs are added. Given their unique textures and structures, various kinds of baked products reacted to SCGs in different ways. SCGs enhanced the taste and fragrance of cookies, which are denser and less moist than bread or cakes. When assessing from an economic viewpoint, it is cost-effective to recycle SCGs, and this significantly assists in the minimization of the environmental footprint of the food industry and the reduction in waste. According to this work, the type of coffee was not specified in some studies—and some studies employed small sample sizes—which potentially limits the applicability and robustness of their findings.

## Data Availability

Not applicable.
